# Real-Time Optical Tracking of Protein Corona Formation on Single Nanoparticles in Serum

**DOI:** 10.1021/acsnano.3c05872

**Published:** 2023-10-06

**Authors:** Mathias Dolci, Yuyang Wang, Sjoerd W. Nooteboom, Paul Eduardo David Soto Rodriguez, Samuel Sánchez, Lorenzo Albertazzi, Peter Zijlstra

**Affiliations:** † Department of Applied Physics and Science Education, Eindhoven University of Technology, 5600 MB Eindhoven, The Netherlands; ‡ Institute for Complex Molecular Systems, Eindhoven University of Technology, 5600 MB Eindhoven, The Netherlands; § Department of Biomedical Engineering, Eindhoven University of Technology, 5600 MB Eindhoven The Netherlands; ∥ Departamento de Física, Universidad de la Laguna, C/Astrofísico Francisco Sánchez, s/n, E-38203 Tenerife, Spain; ⊥ Institute for Bioengineering of Catalonia (IBEC), The Barcelona Institute for Science and Technology (BIST), Baldiri Reixac 10-12, 08028 Barcelona, Spain; # Institució Catalana de Recerca i Estudis Avançats (ICREA), Passeig de Lluís Companys, 23, 08010 Barcelona, Spain

**Keywords:** Protein Corona, Plasmonic Nanoparticles, Dielectric Nanoparticles, Optical Microscopy, Single Particles

## Abstract

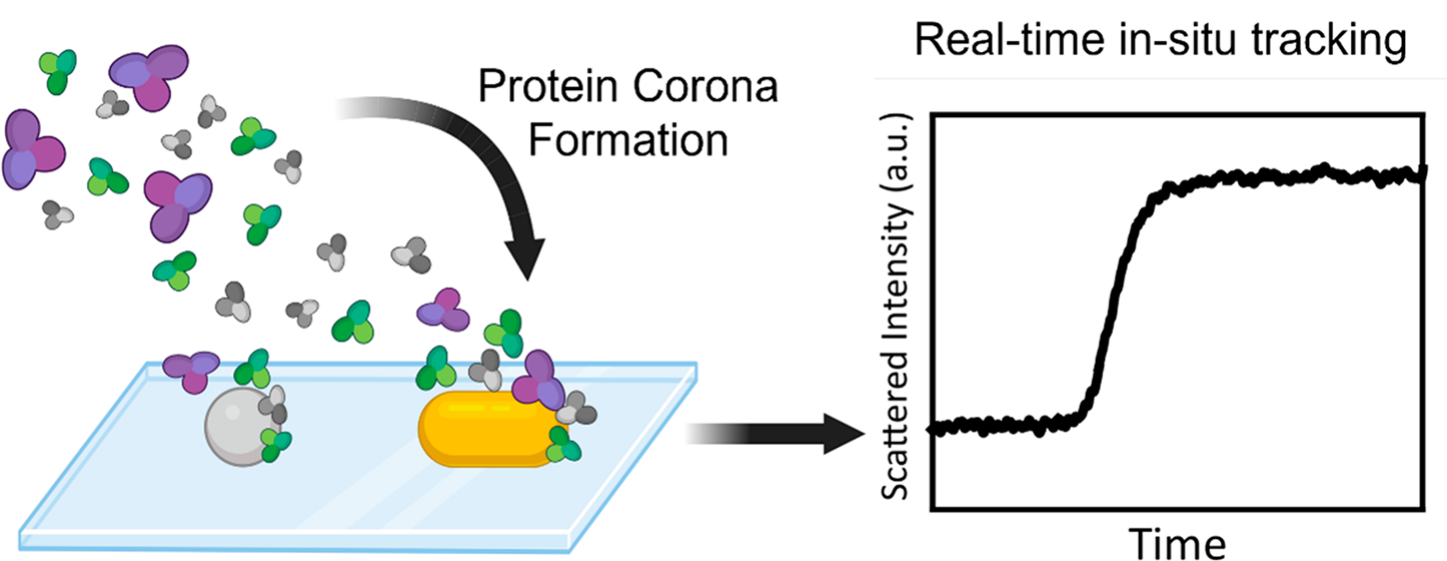

The formation of a protein corona, where proteins spontaneously adhere to the surface of nanomaterials in biological environments, leads to changes in their physicochemical properties and subsequently affects their intended biomedical functionalities. Most current methods to study protein corona formation are ensemble-averaging and either require fluorescent labeling, washing steps, or are only applicable to specific types of particles. Here we introduce real-time all-optical nanoparticle analysis by scattering microscopy (RONAS) to track the formation of protein corona in full serum, at the single-particle level, without any labeling. RONAS uses optical scattering microscopy and enables real-time and in situ tracking of protein adsorption on metallic and dielectric nanoparticles with different geometries directly in blood serum. We analyzed the adsorbed protein mass, the affinity, and the kinetics of the protein adsorption at the single particle level. While there is a high degree of heterogeneity from particle to particle, the predominant factor in protein adsorption is surface chemistry rather than the underlying nanoparticle material or size. RONAS offers an in-depth understanding of the mechanisms related to protein coronas and, thus, enables the development of strategies to engineer efficient bionanomaterials.

Nanomaterials have been increasingly used in the fields of biomedicine and biosensing.^1^ However, a key challenge toward their use is the interaction of such materials with proteins contained in biological fluids, resulting in the formation of a protein coating on their surface, referred to as protein corona (PC). The formation of PCs leads to the alteration of the physicochemical properties of nanomaterials which can result in reduced performances and thus constitutes a significant issue for targeted applications.^2−6^ Understanding the mechanism of PC formation is thus essential to control the behavior of nanoparticles (NPs) and their fate in biological fluids.^7−10^

The formation of PCs is a complex process that is influenced by the properties of the protein (protein charge, hydrophobicity, size, conformation) and by the properties of the nanomaterial (nanoparticle size, shape, functionalization).^11^ Numerous advances in experimental techniques have led to a better understanding of the influence of some of these factors on PC formation.^12−16^ Among the characterization techniques employed, some rely on changes in size and surface charge during the formation of the PC including dynamic light scattering (DLS),^17^ fluorescence correlation spectroscopy (FCS),^18−20^ UV–visible spectroscopy,^21,22^ and gel electrophoresis.^23,24^ In addition, mass spectrometry (MS),^25,26^ circular dichroism (CD),^27,28^ and Fourier transform infrared spectroscopy (FT-IR)^29,30^ enabled the identification and quantification of proteins in the corona. However, as most techniques are not in situ, they result in long processing times, molecular biases in purification processes, and loss of single-particle information. This loss of information might result in contradictory studies and increases the complexity of understanding the mechanisms of PC formation.^31^

Characterization by in situ techniques, on the other hand, eliminates the purification steps that can induce a modification in the composition of the PC. Moreover, they often provide real-time information on the formation of the PCs and thus allow to follow the evolution of the nanobio interface.^32^ The study of the dynamics of corona proteins thus provides valuable access to the protein affinity for the NPs and their ligands. Several studies have provided insights into the mechanisms of PC formation by measuring the affinity constants using isothermal titration calorimetry,^33−35^ quartz crystal microbalance,^36,37^ biolayer interferometry,^38^ surface plasmon resonance (SPR),^23^ circular dichroism,^39^ depletion methods,^40,41^ and electrophoresis.^42^

Since most of these techniques are based on ensemble measurements, they fail to account for the heterogeneity between particles and are prone to particle aggregation in the suspension during PC formation.^43^ Information collected at the single particle level can reveal distributions of behavior that cannot be observed in ensemble measurements. Because aggregates of particles can be distinguished from monomers, single particle analysis is poised to become a major asset in the study of the PC and its heterogeneity.^44^

Despite the need to investigate NP-protein interactions, only a limited number of studies have focused on the in situ formation of PCs at the single-nanoparticle level. For instance, the direct visualization of individual proteins formed on the surface of silica NPs has permitted their quantification and localization using stochastic optical reconstruction microscopy (STORM).^45−47^ These works have revealed the heterogeneous formation of PCs as well as the influence of nanoparticle surface chemistry on their composition. The use of stimulated emission depletion microscopy (STED) has helped distinguish specific structural features of the PCs depending on the geometry of the NPs.^48^ Rotational diffusivity was also used to track real-time PC formation on gold nanorods.^49^ This study reported valuable thermodynamic parameters of protein adsorption at the single NP level. The work of Tan et al. investigated the composition of PCs on fluorescent nanoparticles by discriminating soft and hard corona using a real-time 3D single particle tracking technique (3D-SMART).^50^ This method enabled the real-time tracking of a fluorescent particle without tethering it to a surface, yet required fluorescent labeling. Such labeling is time-consuming, not possible in the case of complex samples like serum, and may result in biases with respect to the labeled species. Ideally protein-corona formation is probed on many single particles in parallel, in real-time, and in situ, without requiring fluorescent labeling of the protein or particle.

Here we introduce a real-time all-optical nanoparticle analysis by scattering microscopy (RONAS) to track the formation of PC in situ, at the single-particle level, without the need for any labeling. RONAS exploits the sensitivity of a particle’s scattering cross section to its local environment, enabling the label-free and all-optical tracking of PC formation on hundreds of particles in parallel directly in serum. We use RONAS to study the amount, affinity, and kinetics of the PC on gold and silica nanoparticles with sizes ranging from 50 to 200 nm. Despite an unexpectedly strong particle-to-particle heterogeneity, the corona consists of a submonolayer of comparable average thickness for both gold and silica particles with an amine-rich coating. We find that the porosity of the particle strongly affects the kinetics of PC formation, which is attributed to an interplay between rapid surface adsorption and the slow internalization of proteins in the nanoparticle’s pores. The presented method gives promising prospects for studying the effects of particle shape, size, material, and coating on single nanoparticles in high throughput and in real-time (Figure 1a).

**Figure 1:**
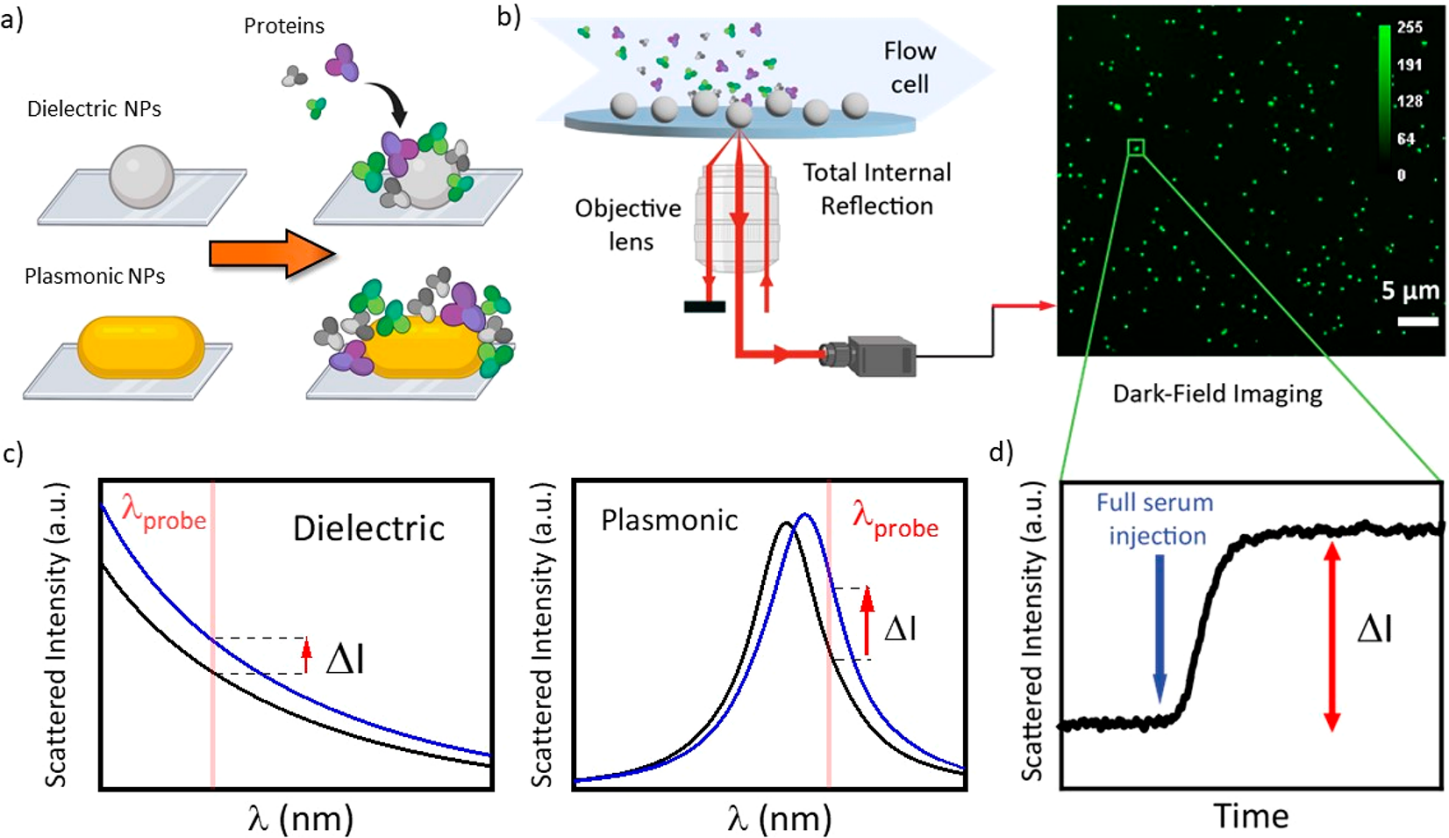
(a) Schematic representation of the workflow wherein single particles are probed in real-time in a fluidic system during the formation of PC. (b) Schematic of the RONAS setup using total internal reflection excitation to perform single-particle scattering microscopy (left). A flow cell is mounted on top of the sample to enable fluid exchange during real-time probing of the nanoparticles immobilized on a coverslip. The light scattered by the nanoparticle is directed toward a CMOS camera to enable investigation of many particles in parallel (right). (c) Simulation showing the scattering cross section of dielectric and plasmonic nanoparticles before (black curve) and after (blue curve) protein adsorption at their surface. At the probe wavelength, the scattering cross section increases (Δ*I*) because of the increase of the local refractive index due to PC formation. (d) Example of data from (b) showing the trace of the scattered intensity on a single nanoparticle.

## Approach

Metallic and dielectric nanoparticles are widely used in the field of biomedicine as theranostic agents and benefit from their plasmonic properties^51^ and high biocompatibility,^52^ respectively. Their high scattering cross section allows for straightforward optical detection at the single-particle level.^53−55^ Hence, optical detection by scattering is a perfectly suitable tool for the investigation of protein corona since it provides in situ and real-time monitoring at the single particle level and does not suffer from blinking and bleaching like fluorescent methods do. The increase of the refractive index near the particle’s surface (induced by the binding of biomolecules for example) leads to a change in their scattering cross section (Figure 1c). For plasmonic nanoparticles, the plasmon resonance red shifts upon biomolecular binding,^56^ whereas for nonresonant dielectric nanoparticles the adsorption of PC results in an increase in their Rayleigh scattering cross section due to an effective increase in particle size. The formation of a protein corona on both dielectric and plasmonic nanoparticles can therefore be tracked by an integral approach that monitors changes in single-particle scattering properties.

To probe these changes in the scattering cross section of the nanoparticles, dark-field microscopy was used. Regular dark-field condensers provide straightforward implementation, but their transmission geometry results in a strong background signal due to scattering by the serum components. Instead, a total internal reflection (TIR) configuration was implemented to generate an evanescent wave propagating only in the vicinity of the microscope coverslip, thereby negating the background signal from the serum (Figure 1b). This specificity, based on probing only the particles without being affected by background noise, enables the study of interactions in complex environments. Moreover, the method relies on a change in the particle scattering and allows the investigation of all types of particles regardless of the materials used. The RONAS method offers the opportunity to study a wide range of systems, provided that their scattering cross section is high enough to provide sufficient a signal-to-noise ratio in the single-particle microscope. This provides access to the most used particle sizes with diameters above ∼40 nm for metal particles and ∼80 nm for dielectric ones. However, label-free interferometric techniques have recently been reported that extend the accessible size regime to very small particles (<5 nm).^57,58^

Three types of nanoparticles were investigated with different sizes, shapes, and material to demonstrate the generality of RONAS: (i) gold nanorods (20 nm-diameter and 75 nm-length) coated with cysteamine (AuNRs), (ii) 150 nm diameter silica nanospheres coated with APTES (SiO_2_-smooth), and (iii) 200 nm diameter mesoporous silica nanospheres coated with APTES (SiO_2_-porous) obtained by a synthesis described previously.^59^ A more detailed description of the synthesis and functionalization of the porous nanoparticles can be found in the Materials and Methods section. Due to the nature of our label-free approach, it is however not restricted in terms of particle geometry, and different shapes and types of materials can be studied. The nanoparticles were immobilized on a coverslip by spin-coating which results in a sparse distribution allowing for the imaging of many single nanoparticles in parallel (Figure 1b). A microfluidic channel was mounted on top of the sample to enable fluid exchange during acquisition. RONAS therefore allows for the real-time investigation of protein adsorption on the same set of single nanoparticles under varying serum dilutions and enables the investigation of adsorption as well as desorption kinetics.

Changes in the scattering cross section of single particles were probed by using a supercontinuum white light source filtered with a bandwidth of 10 nm around a center wavelength of 600 nm (for silica) and 780 nm (for AuNRs), respectively. Real-time measurements were performed with injection of undiluted fetal bovine serum (FBS) with a constant flow rate (0.25 mL/min) leading to the formation of a protein corona that was monitored in real-time at the single-particle level (Figure 1d). Time-traces were extracted by 2D Gaussian fits to the point spread functions for each time point to determine time-dependent changes in the scattered intensity.

RONAS is capable of monitoring PC formation dynamically at a video rate and for arbitrary times. For the AuNRs, the change in the scattered intensity, caused by the binding of the proteins, can be positive or negative (Figure S1) depending on the position of the probe wavelength with respect to the plasmon resonance of each single particle.^60^ To enable quantitative comparison, we developed a numerical algorithm that converts the change in scattered intensity into a plasmon shift. This algorithm uses the measured scattering spectrum for each individual particle to obtain the plasmon shift in real-time (see the Materials and Methods, and Figure S2). RONAS provides millisecond temporal resolution without the need for a spectrometer, allowing many particles to be compared in parallel as a function of time.

## Results and Discussion

### In Situ Measurement of PC Formation on Single Nanoparticles

The scattering cross sections of nanoparticles (dielectric and plasmonic) are highly sensitive to local changes in the refractive index. Therefore, the injection of undiluted FBS induces a change in the scattering cross section due to two mechanisms: (1) the formation of a PC on the surface of the particles and (2) the possible changes of the bulk refractive index. These effects were decoupled by first injecting a mix of PBS and ethylene glycol whose refractive index matches that of FBS, followed by the injection of undiluted FBS. The refractive index of FBS was determined to be *n* = 1.3417 by refractometry, equivalent to a 1:11 mixture of ethylene glycol and PBS. This multistep procedure not only enables the decoupling of surface and bulk effects but also provides an internal calibration of the refractive index sensitivity of each single particle.

**Figure 2:**
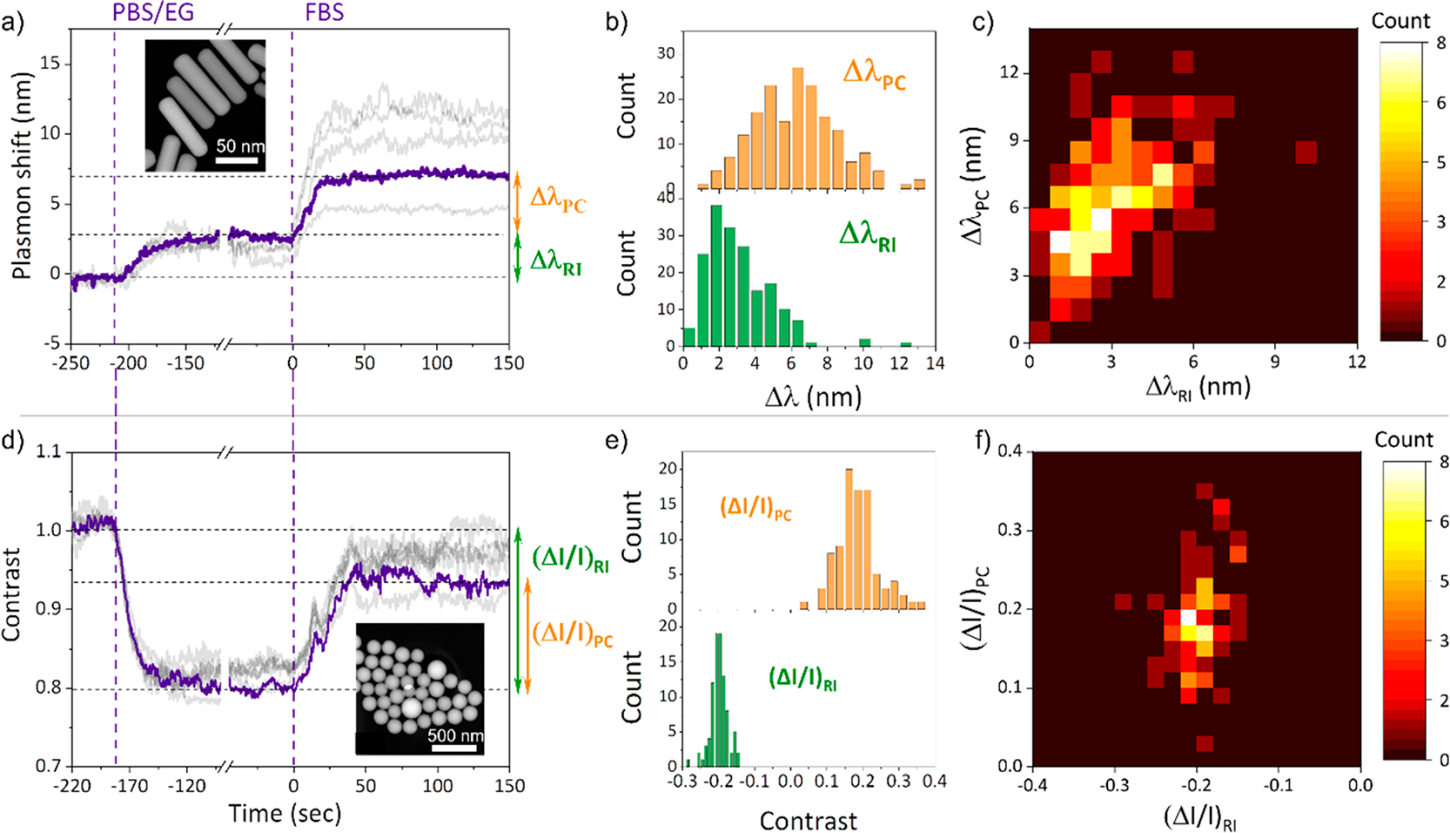
(a) Timetrace of the plasmon shift of different single nanorods in the field of view. The first vertical dashed line corresponds to the injection of the PBS/EG buffer to match the bulk refractive index of FBS, and it gives rise to a red shift of the plasmon resonance (Δλ_RI_). The second dashed line corresponds to the injection of FBS that gives rise to a second plasmon shift (Δλ_PC_) due to PC formation. A TEM image of the gold nanorods is shown in the inset. (b) Histogram of the plasmon shift due to PC (upper graph) and due to the change of bulk refractive index (lower graph). (c) Heatmap showing the correlation between the plasmon shifts due to index matching and PC formation. (d) Timetrace of the contrast for different single silica nanoparticles in the field of view. The vertical dashed lines are identical to (a). A TEM image of the silica nanoparticles is shown in the inset. (e) Histogram of the contrast due to PC (upper graph) and due to the change of bulk refractive index (lower graph). (f) Heatmap showing the correlation between the contrast due to index matching and PC formation.

For the plasmonic particles, both injections lead to a red shift in the plasmon resonance (Δλ_RI_ and Δλ_PC_, respectively); see Figure 2a. The additional shift induced by injection of the FBS can therefore be assigned to the protein adsorption only. It is important to notice that saturation in the plasmon shift after injection of FBS is rapidly reached indicating the formation of a protein layer on the surface on short time scales (the characteristic time scales will be discussed later). Furthermore, after replacing serum with the index matching buffer the scattered intensity remained stable, indicating that no detectable protein desorption takes place on time scales of tens of minutes (Figure S3a). This behavior suggests that this layer is stable toward buffer exchange, which is characteristic of proteins described as a “hard corona” where exchange of proteins in the PC is slower than typical experimental time scales.^61^

Single-particle optical imaging provides insight into particle-to-particle differences in the PC. For plasmonic particles, however, the plasmon shift induced by a certain PC layer thickness may vary from particle-to-particle due to differences in refractive index sensitivity.^62^ Using the buffer exchange at the start of each experiment, we therefore also quantify the refractive index sensitivity of each single particle. The plasmon shifts due to bulk index changes (Δλ_RI_) and PC formation (Δλ_PC_) were extracted for all the AuNRs in the field of view of the microscope and plotted as histograms (Figure 2b). The histogram of the shifts due to PC formation reveals a wide distribution of red shifts (shifts ranging from 1 to 13 nm).

A heatmap displaying the distribution of shifts due to PC formation versus shifts due to refractive index change is shown in Figure 2c. A correlation is observed between Δλ_RI_ and Δλ_PC_ (with a Pearson correlation coefficient *r*′ = 0.60), which is largely caused by differences in the aspect ratios of the AuNRs. The dispersion observed on the *x*-axis corresponds to the different sensitivities of the AuNRs to a change in the bulk refractive index. A vertical cross-section in the heat map thus indicates heterogeneity in terms of the adsorbed protein mass which is larger than 1 order of magnitude across all particles. This necessarily implies a large distribution in the number and/or species of protein on the surface of these AuNRs. We then normalize the plasmon shift due to PC formation to the bulk index sensitivity of each single particle to obtain a metric that is not biased by bulk index sensitivity. In addition, the bulk index sensitivity does not correlate strongly with the shift due to PC formation, indicating that the heterogeneity observed originates from particle-to-particle variability in the amount of PC adsorbed. This is also supported by the timetraces in Figure 2a, which illustrates several particles that exhibit the same red shift due to the buffer exchange but display a very different shift upon PC adsorption.

Boundary element method simulations were performed to evaluate the effective thickness of the protein layer (Figure S4). These simulations were carried out for different protein layer thicknesses and different refractive indices of the protein layer (corresponding to realistic values of the effective index of proteins found in blood).^63^ Given that the average plasmon shift due to PC formation is Δλ_PC_ = 6.1 nm, this would be equivalent to an effective layer thickness between 1.4 and 4.3 nm. Despite the fact that FBS is a complex medium with proteins of different sizes and refractive indices, these values suggest the formation of an incomplete monolayer particularly because the most abundant proteins in the blood (albumin and globulin) are substantially larger (dimensions of ∼7 and ∼10 nm respectively). It should be stressed, here, that the interaction of proteins with the particle surface might induce a conformational change in the protein structure.^64,65^ Unfolding of the protein may thus occur, effectively resulting in a thinner corona layer due to spreading. This submonolayer formation might hence be partially attributed to the unfolding or spreading of proteins, which could in the future be investigated by correlated single-molecule fluorescence measurements as in ref (76).

This observation contrasts with part of the literature that observes multilayers on nanoparticles of similar sizes.^66,67^ Lin et al, for example, observed effective thicknesses in excess of 13 nm for a similar system (gold nanorod in a protein mixture) and attributed this to the formation of multilayers.^49^ However, it is important to note that analysis techniques based on the measurement of size or mobility of NPs in solution (such as DLS) cannot distinguish between PC formation and particle clustering.^44^ RONAS is insensitive to aggregation and, therefore, accounts for PC formation exclusively.

In the case of silica nanoparticles, the intensity contrast is monitored, which is defined as the normalization of the signal to the initial intensity measured during the first 60 s in PBS. The injection of the index-matching PBS/EG solution reduces the RI-contrast between particle and medium, and therefore reduces the scattering cross section (Figure 2d). Upon injection of blood serum, on the other hand, the formation of PC effectively increases the diameter of the nanoparticle and enhances their scattering cross section. Similarly to AuNRs, no decrease in the scattered intensity is observed after rinsing, indicating that the protein layer consists of a hard corona (Figure S3b).

The distribution of contrasts for protein adsorption is broader than the one due to the bulk refractive index change (Figure 2e). The low contrast variation (*ΔI*/*I*)_RI_ for dielectric particles is because their intrinsic sensitivity to refractive index changes is only slightly dependent on their size. This narrow contrast distribution (with a coefficient of variation CV = 0.13) is therefore expected and can be related to the size heterogeneity of the silica particles. On the other hand, the coefficient of variation of the contrast distribution due to the PC formation (CV = 0.31) is much higher and reflects a high level of heterogeneity in the number of adsorbed proteins on the surface of the nanoparticles. This behavior can be directly visualized in a heat map (Figure 2f) exhibiting a small dispersion upon solvent change but a large dispersion due to the PC formation. This is reflected with the Pearson correlation coefficient, which is way lower than for the gold nanorods (*r*′ = 0.28). The influence of the particle size on the heterogeneity in the protein layer has already been widely demonstrated.^68,69^ However, these changes in size lead to a modification in the radius of curvature, which has a strong influence on the adsorption of proteins and is more preponderant for small size nanoparticles. It is more likely that the heterogeneity in PC that we observe originates from heterogeneities in surface chemistry between the particles (in terms of local charges and polarity).

Similar to the case for AuNRs, simulations were performed to gain insight into the effective thickness of the protein layer. Mie theory was used to simulate the absorption of a protein layer (Figure S5) corresponding to the experimentally observed average contrast enhancement (18%). This contrast would be equivalent to an effective thickness between 1.5 and 4.5 nm (depending on the refractive index chosen for the protein layer). The same submonolayer thickness was found for AuNRs, suggesting that PC formation is largely independent of material and shape of the underlying nanoparticles but more sensitive to the surface chemistry. This conclusion seems rather unexpected in view of the literature, where shape, size, and materials appear to have a significant influence on PC formation. It may be that surface chemistry is a more critical factor than nanoparticle core material, but additional measurements with different ligands should be carried out to confirm this hypothesis.

RONAS provides valuable information on the kinetics and amount of PC at the single-particle level. This has revealed that large particle-to-particle differences underlie the PC adsorption process, while reversible PC adsorption has not been observed on the particles that we investigated. The compatibility of the approach with any particle size and shape (as long as it scatters sufficiently) revealed that the PC and its heterogeneity are not dictated by particle shape or material but are likely dominated by heterogeneous surface chemistry. Coupling RONAS with another analytical method would provide a complete understanding of corona proteins in terms of both formation kinetics and composition. Gel electrophoresis^70^ or mass spectroscopy^26,71^ are commonly used methods to study the composition of PCs; however, these ensemble methods result in the loss of single particle information. In another way, techniques providing information on the composition at the scale of the individual nanoparticle can be combined with the RONAS method using correlation microscopy. Scattering methods offer the advantage of being compatible with other imaging methods, such as fluorescence. This offers the possibility of measuring several tagged proteins on the same particles, as was achieved with STED microscopy^48^ and should be envisioned in the near future.

### Effective PC Affinity

**Figure 3:**
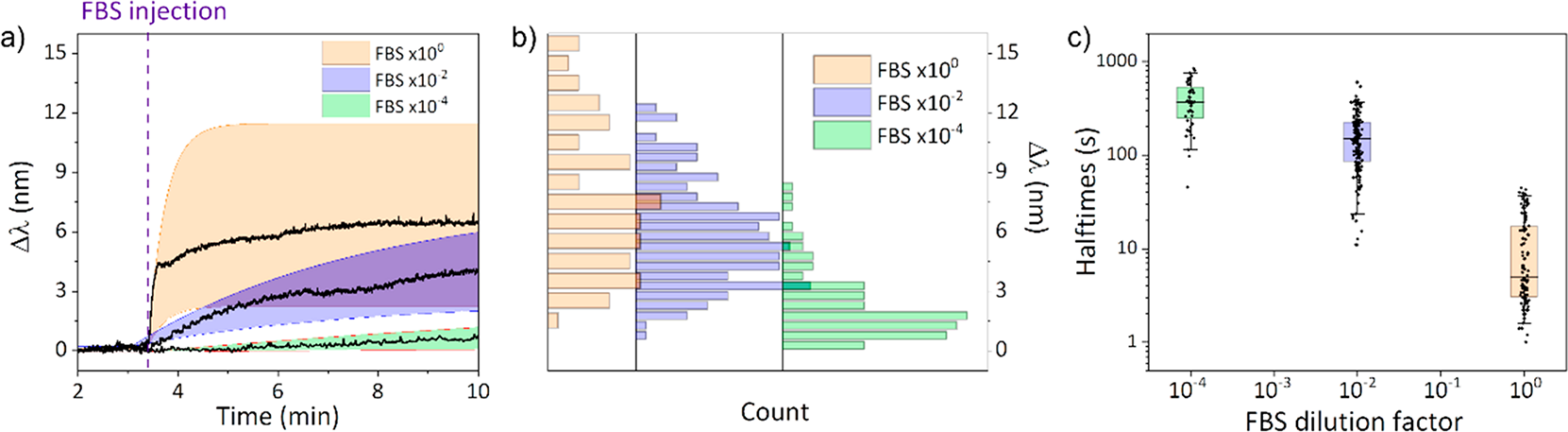
(a) Timetrace showing the average plasmon shift for all nanoparticles in the field of view after injection of FBS at different dilutions. The vertical dashed line corresponds to the time of the injection. The shaded areas correspond to the standard deviation of the plasmon shift over the entire sample. (b) The corresponding distribution of the plasmon shifts for the same data as in (a). (c) Bee-hive plot of the single-particle half-times versus the FBS concentration.

PCs are not static but are subject to a dynamic process of single-molecule adsorption and desorption. This results in, e.g., the Vroman effect wherein low affinity interactions are gradually replaced by high affinity interactions. It is therefore important to extract kinetic parameters quantitatively to gain insight into these adsorption mechanisms. The adsorption of protein was thus monitored at different FBS dilutions, shown in Figure 3a. For undiluted and 10-fold diluted (data not shown) serum, a rapid saturation within seconds was observed. For higher dilutions of FBS, a gradual increase was observed which did not reach saturation over the measurement duration as confirmed with the plasmon shift distributions (Figure 3b). To assess kinetics at the single-particle level, the half-times, which correspond to the time at which half the plasmon shift was measured, are plotted as a function of the FBS dilution factor (Figure 3c). The relatively short half-times (on the order of ten s) for the full FBS are at least 40 times shorter than for the dilution investigated. This kinetics may result from a combination of two mechanisms: the slower association of proteins and the Vroman effect, which effectively leads to a gradual increase in the affinity between the particle and the PC. These data should be considered together with the insights gained in recent years on the importance of the adsorption time of the proteins. Indeed, it has been shown that proteins tend to rapidly form a weakly bound layer to the NPs, but as adsorption time increases, these weak interactions are progressively replaced by stronger (and eventually irreversible) interactions.^72,73^ In addition, at low protein concentrations, the slow association rate enables the early adsorbed proteins to have more time to rearrange into a more stable configuration. These configurations, which may be structural relaxations, provide reduced space for subsequent proteins, resulting in a more compact PC structure.^44^ Taken together, these mechanisms explain the gradual plasmon shift for high dilution factors. In addition, a higher degree of heterogeneity in the distribution of half-times for full serum (CV = 1.03) is observed compared with that of the most diluted sample (CV = 0.50). This reduced heterogeneity as well as the lower total shift value for lower FBS concentrations suggest that under potential rearrangements, the configuration of proteins results in thinner layers driven by protein relaxation.^44^

The affinity of the PC is an important metric for the underlying mechanism and interaction strength between the PC and the NP surface.^74,75^ Real-time studies have enabled the extraction of the dissociation constant *K*_D_, a key thermodynamic parameter that reflects the affinity of proteins for NPs. Hühn et al. reviewed the affinities measured by different methods and for different nanoparticles and proteins.^75^ The values found for *K*_D_ are highly variable (ranging from mM to pM), and too few systematic studies have been conducted to draw firm conclusions. One piece of information that has been extracted is the influence of surface charge, where moderately positively charged NPs appear to show the highest affinity.^75^ We demonstrated above that RONAS tracks protein corona formation in real-time on single nanoparticles, which we now apply to investigate the affinity of the PC at the single-particle level.

**Figure 4:**
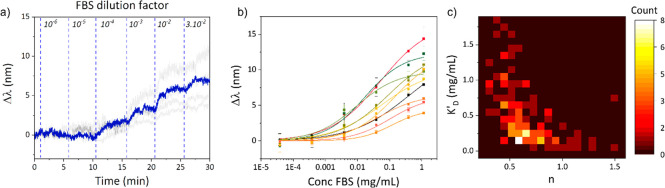
(a) Typical timetraces of a single nanorod with sequential injection of FBS at increasing concentrations. (b) Dose–response curves of several nanorods fitted with a Langmuir-Hill model. (c) Heatmap showing the correlation between the apparent dissociation constant and the Hill coefficient for each single nanoparticle.

Figure 4a shows different timetraces corresponding to several single gold nanorods in the field of view upon sequential injection of increasing concentrations of FBS. Higher serum concentrations were not used in order to avoid a change in the bulk refractive index that is difficult to correct for in a single workflow that contains multiple serum dilutions. Although these conditions differ from those of full serum, the kinetics observed for an undiluted sample and a sample diluted with the lowest dilution factor showed similar kinetics. Adding higher concentrations to the dose response curve will, therefore, not change the conclusion. A red shift can be observed from the highest dilution of FBS injected for certain nanoparticles, while other particles accumulate PC only at 10- to 100-fold lower serum dilutions. The values of the shift were extracted and plotted for each concentration of FBS (Figure S6). These data were extracted for each nanoparticle in the field of view and fitted with the following Langmuir-Hill model (Figure 3b):
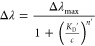
1where Δλ_max_ represents the maximum resonance shift induced by the PC, *K*_D_^′^ the apparent affinity corresponds to the serum concentration at which the change in scattering cross section of the nanoparticles is 50% of the maximum shift, *n*′ is the apparent Hill coefficient and *c* is the effective concentration of protein introduced. The Langmuir-Hill model is the most widely used to depict protein adsorption due to the interaction between proteins once adsorbed on the surface of the NPs. It is important to mention that here this model is simplistic since it is suitable for the adsorption of a single protein via a well-defined biochemical interaction. In the present case, there are thousands of different proteins in the blood serum that adsorb via different mechanisms and may be displaced due to the Vroman effect. For this reason, an effective Hill coefficient and dissociation constant were extracted, enabling us to study particle-to-particle differences.

The dose–response curves for different particles (Figure 4b) clearly illustrate the heterogeneity, as some particles reach saturation for concentrations below 1 mg/mL, while others do not saturate even for the highest concentrations. The data collected from the fitted curves were used to extract the values of *K*_D_^′^ and *n*′.

The heatmap of the apparent dissociation constants and apparent Hill coefficient (Figure 4c) shows a broad distribution among the nanoparticles in the sample. The *K*_D_^′^ shows a particle-to-particle heterogeneity across 2 orders of magnitude. Nearly all of the analyzed nanoparticles display a value of *n*’ lower than 1, suggesting an anticooperative behavior during the formation of the protein layer. It is noteworthy that a degree of correlation exists between the effective Hill coefficient and dissociation constant, where particles with low affinity (high *K*_D_^′^) correspond to the most anticooperative particles (low *n*’). Such anticooperative behavior that is correlated to the affinity indicates that the particles initially accumulate a sparse coating that slows subsequent protein adsorption. This results in an anticooperative adsorption of proteins that eventually leads to the formation of a submonolayer of PC, in agreement with our previous observations.

When examining the literature, it becomes clear that it is not trivial to expect a specific behavior since values of *n*′ are reported to range from less than 1 to more than 4 (indicating in the latter case a very strong cooperativity during protein adsorption).^75^ It is however difficult to compare reported systems, presenting nanoparticles of different sizes, morphologies, compositions, and with different surface chemistries. Compared to other methods, however, RONAS is immune to aggregation and labeling artifacts, which results in a generic anticooperative behavior in undiluted serum.

### Effect of Particle Porosity

The versatility offered by RONAS has shown that materials of different natures (dielectric and plasmonic) can be studied with an integral method. This addresses the need in the field where single-particle studies are often limited to very specific systems (e.g., use of localized surface plasmon,^76^ use of anisotropic particles,^49^ or fluorescent labeling^46,50^). Nevertheless, the opportunity to study systems with different functions and structures is equally important. For example, Piloni et al. showed that surface roughness could influence the formation of PCs as well as their cellular uptake.^77^ For this reason, we employed RONAS to compare PC formation on smooth and porous materials to investigate in real-time the effect of porosity on the amount and kinetics of protein adsorption.

**Figure 5:**
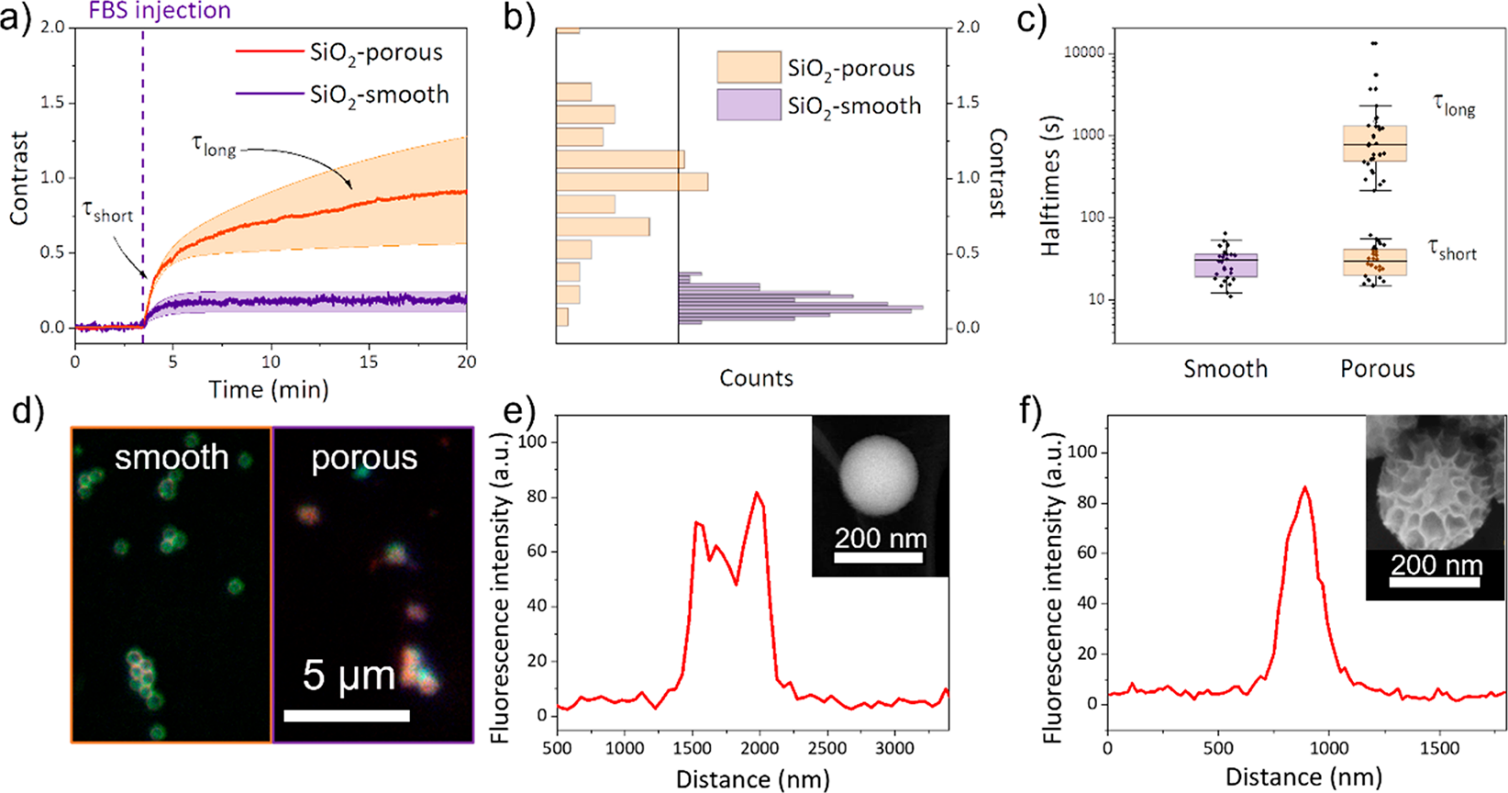
(a) Averaged timetraces during PC adsorption on SiO_2_ nanoparticles with a smooth surface (purple line) and a porous structure (red line) upon exposure to FBS. The vertical dashed line corresponds to injection of 0.05× FBS. The shaded areas correspond to the standard deviation of the contrast over the entire sample. The contrast has been normalized to the initial scattered intensity (3 × 10^5^ camera counts for the smooth NPs and 4 × 10^5^ camera counts for the porous NPs). (b) Corresponding histograms of the contrast for the two samples. (c) Bee-hive plot of the time constants extracted from exponential fits to the timetraces of single particles. (d) STED image of both types of nanoparticles with a corona of fluorescently labeled BSA. Line profile across a single nanoparticle imaged for (e) smooth and (f) the porous nanoparticles. The inset shows a representative TEM image of the particles.

Silica nanoparticles of 200 nm diameter with high porosity (KCC-1 type silica with the porosity defined as the spacing between fibers)^59^ were compared with smooth particles. A rapid increase within a few minutes was observed in contrast to the averaged time traces for both types of nanoparticles after the injection of 20-fold diluted blood serum (Figure 5a). However, the average contrast of porous particles is nearly 7 times higher than that of smooth particles. The contrast distribution (Figure 5b) is also broader (CV = 0.49 for the porous NPs and CV = 0.31 for the smooth NPs) indicating a higher heterogeneity in PC formation. The difference in size between the two types of nanoparticles is insufficient to account for such a difference in contrast (the size of porous NPs being only 30% larger than the smooth ones). The large increase in the contrast is therefore attributed to the uptake of protein in the pores, thus filling in the wrinkles and significantly increasing the refractive index of the nanoparticles leading to a higher scattered intensity. The broader distribution in the contrast can hence be explained by the heterogeneity of the porous structure itself as well as by the heterogeneous adsorption of the proteins.

A second observation can be established on the adsorption kinetics: the porous NPs do not show saturation in the contrast even after 15 min of serum incubation. While previously halftimes were well suited to the study of adsorption kinetics for a single process, here, the time scales for which PCs form can be quantified by fitting exponential functions. In the case of the smooth particles, the data can be well fitted by a single exponential function, whereas the porous silica particles were fitted with a double exponential (see eqs 2 and 3 in the Materials and Methods section).

The distributions of time constants (Figure 5c) indicate that both particle types show a fast time component on the order of 1 min, whereas only the porous NPs present this secondary adsorption from tens of minutes to several hours. We therefore attribute the short time scale to PC formation on the exposed outer surface of the particles, whereas the slower time scale observed for porous particles is attributed to the slower diffusion and subsequent binding of protein in the nanoparticle pores. To further confirm this mechanism, we performed super-resolution microscopy on the two particle types (Figure 5d). To this end, bovine serum albumin (BSA), which is the most abundant protein in FBS, has been labeled with Star Red dye before incubation with the nanoparticles (see details in the Materials and Methods section). While the smooth NPs present a distribution of PCs on their surface (as shown by the two peaks and the central dip in the profile (Figure 5e), the porous NPs exhibit a more homogeneous distribution, indicating the presence of proteins inside their core (Figure 5f).

The proteins gradually fill the wrinkles in the nanoparticles, as previously observed, with smaller proteins tending to migrate more easily inside the structures.^48^ Furthermore, the significant differences observed in the long adsorption times (τ_long_) indicate considerable heterogeneity in the porosity of the NPs resulting in heterogeneous protein corona formation kinetics. These results offer the opportunity for the real-time study of PC formation on exotic nanoparticles with more complex structures and may aid in the understanding of uptake mechanisms of, e.g., drugs in particle-based carriers.

## Conclusion

To summarize, RONAS enables label-free imaging of single nanoparticles to study real-time protein corona formation on the surface of metal and dielectric nanoparticles with different porosities. We found a strong heterogeneity of the PC in undiluted blood serum, with adsorbed protein masses varying across more than 1 orders of magnitude. Furthermore, single-particle dose–response curves revealed anticooperative behavior, consistent with the initial rapid association of the protein providing a sparse coating that slows down subsequent protein adsorption. The study of porous particles with the real-time and in situ measurement revealed a multiphase behavior not previously observed until now. This suggests that fast adsorption of proteins is followed by slower migration of proteins into the porous structures, which was confirmed by super-resolution microscopy.

The simplicity of RONAS suggests its usefulness for systematic and high-throughput studies that are still needed to further understand the PC formation mechanisms on bionanoparticles. The optical interrogation we propose is generic and enables the study of PC formation in situ and in real-time on a wide variety of particles ranging from metallic to dielectric with different sizes, structures, and surface chemistry. In addition, the simplicity of the setup and integration with microfluidics facilitate high throughput measurements and automated screening. In addition, combining RONAS with other single particle analysis methods would provide a full characterization of the formation mechanisms and composition of the protein layer. Ultimately, particles as small as several nanometers which are commonly used for theranostic applications could be interrogated by using interferometric microscopy.^78^

## Materials and Methods

### Porous Silica Nanoparticle Synthesis

For the synthesis, a modified method was used based on that reported by Bayal et al.^79^ Briefly, CTAB (1 g), water (30 mL), and urea (600 mg) were mixed, and a mixture of tetraethyl orthosilicate (TEOS) 6 mmol and cyclohexane (30 mL) was dropwise added to form a lamellar phase of CTAB producing a wrinkled surface on silica. To stabilize the emulsion, 1-pentanol (1 mL) is dropwise added. The mixture was left for 16 h and refluxed at 70 °C under magnetic stirring. The particles were collected and washed three times by centrifugation and then dispersed in ethanol. To remove the CTAB, the samples were added to a solution of ammonium nitrate (160 mg) in ethanol (60 mL) at 60 °C for approximately 30 min; hereafter, the samples were washed with ethanol and dried in a vacuum. For the functionalization, the particles were dispersed in EtOH (2 mg/mL) and sonicated (10 min). Once well dispersed, APTES (10 μL/mg of particle) was added directly to the solution. The obtained mixture was stirred for 24 h at 50 °C with an end-to-end rotary shaker. Subsequently, the functionalized particles were centrifuged at 7.5 K rpm for 10 min and washed in ethanol (×3) and water (×3), with sonication for 15 min between each centrifugation. Finally, aliquots (0.5 mL) were collected, centrifuged, and air-dried to determine the concentration of the particle suspension.

### Sample Preparation

Glass coverslips (thickness #1.5) were sonicated in methanol for 15 min and dried under a nitrogen flow. The coverslips were rendered hydrophilic by a plasma treatment for 1 min. Then, 10 μL of a suspension of AuNRs (A12-25-780-CTAB, OD 1, NanoPartz) was spin coated onto the cleaned microscope glass coverslip. Excess CTAB was removed by rinsing with methanol, phosphate-buffered saline (PBS), and distilled water. The nanorods were functionalized by incubating in a 10 mM cysteamine (Sigma-Aldrich) aqueous solution. After incubation, the sample was extensively rinsed with mL of water and dried in a nitrogen flow. For the SiO_2_-smooth (150 nm, 5% weight in water, Sigma-Aldrich) and SiO_2_-porous particles, 10 μL of a suspension of silica spheres was directly spin coated onto a microscope glass coverslip, then dried under nitrogen flow.

### Single-NP Dark-Field Spectroscopy

Single-NP spectra were measured by objective-type total internal reflection microscopy on an inverted wide-field microscope (Nikon Ti2). The sample was illuminated through an oil-immersion 1.49 NA objective. The direct reflection was blocked by a beam block, after which the scattered light was projected onto an CMOS camera (Photometrics Prime BSI Express). The scattered intensity as a function of wavelength was obtained by fitting a 2D Gaussian to the point spread function by using custom Python software.

### Data Collection

The illumination was performed using a supercontinuum white-light source (SuperK Compact, NKT Photonics) with an acousto optic tunable filter (AOTF) enabling the spectroscopy of the nanoparticles with a bandwidth of 10 nm and central wavelength ranging from 600 to 840 nm (Figure S2). The choice of the probe wavelength for the real-time measurement was done based the spectroscopy results. Silica particles exhibit Rayleigh scattering and therefore have the largest scattering cross section at shorter wavelengths. Optimal signal intensity also depends on the collection efficiency of the setup, and consequently, the probe wavelength was set to 600 nm, which corresponds to the optimal signal obtained. The largest sensitivity to plasmon shifts for AuNRs is for a probe wavelength at the full-width-at-half-maximum of their longitudinal plasmon resonance. Due to the size dispersion in even the best batches of nanorods, the plasmon wavelength varies from particle-to-particle. For that reason, the wavelength chosen for these nanoparticles was 780 nm (corresponding to the averaged longitudinal plasmon resonance wavelength of the AuNRs used in this study), and the scattered intensity was converted to a plasmon shift using the algorithm described below. The timetraces were extracted using a Python script that fits a 2D Gaussian to the point spread functions for each frame with an integration of 100 ms.

### Conversion of the Change in Scattered Intensity to Plasmon Shift for the AuNRs

For silica particles, we evaluate the data in terms of changes in scattered intensity. For gold particles we present a conversion algorithm that converts the change in scattered intensity into a plasmon shift (see Figure S7 for the quantities which are used in the following derivation). This enables direct comparison of all particles in the measurement, irrespective of their plasmon wavelength, which is not possible if only the scattered intensity is evaluated. The conversion of the intensity scattered by a single nanoparticle into a plasmon shift can be done assuming that the plasmon resonance of a nanorod can be approximated by a Lorentzian function given by
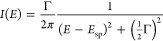
where *E* is the incident photon energy, and *E*_sp_ and Γ are the plasmon energy and line width (measured from the scattered spectrum), respectively. Note that all units are in eV. In a biosensing experiment, this spectrum becomes time dependent due to a time-dependent shift of the plasmon resonance. This can be taken into account by considering *E*_sp_ to be time-dependent due to a shift Δ*E*_sp_(*t*). The plasmon resonance is then given by
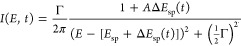


Herein the factor *A* has a negative value and describes the increase in scattering cross section in response to a plasmon red shift (i.e., a decrease in cross section with an increase in plasmon energy). The contrast in an intensity-based experiment is probed using a light source with center energy *E*_p_. We assume that the line width of the source is much narrower than Γ. The contrast is then given by
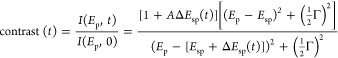


Provided the value of *A* is known (either estimated from the asymmetric shape of a typical s-curve or estimated from a numerical model of the scattering spectrum), this approach can be used to solve analytically for Δ*E*_sp_(*t*). For *A* = 0 we can extract Δ*E*_sp_(*t*) directly:
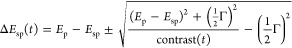
where the plus-sign holds when *E*_p_ < *E*_sp_ and the minus-sign holds when *E*_p_ > *E*_sp_.

For *A* ≠ 0 we first need to rewrite the equation into
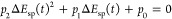
where
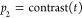

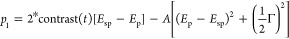

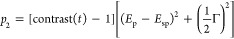


From here, Δ*E*_*sp*_ (*t*) is easily calculated:
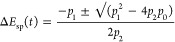
where the plus sign holds when *E*_p_ < *E*_sp_ and the minus sign holds when *E*_p_ > *E*_sp_.

This derivation also assumes that the line width Γ is not affected by the adsorption process. Although this is not necessarily true in all cases, our experimental data show that the line widths of single nanoparticles change by only 4.4 ± 22.9 meV, justifying the approximation that the line width remains constant (Figure S8). The distribution in the histogram is caused by measurement and fitting uncertainties to determine the line width in the spectra.

### Model for Protein Adsorption

The kinetics of protein adsorption is commonly expressed by the Langmuir model as the fraction of surface coverage θ as a function of time *t*.
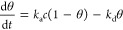


With *k*_a_ and *k*_d_ the association and dissociation constants, respectively, and *c* the concentration of protein in solution. For describing the association of the proteins onto the particles, we neglect the dissociation part because at serum concentrations *k*_a_*c* ≫ *k*_d_. The contrast in intensity measured experimentally  is then directly proportional to the surface coverage, and the function for fitting the smooth silica particle is
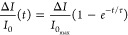
2with  the maximum change in contrast intensity and τ the time constant .

In the case of porous silica, the contrast is fitted with a double exponential where two associations can happen at different time scales (*k*_short_ and *k*_long_):
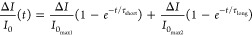
3where τ_short_ and τ_long_ are the short and long time constants, respectively.

### STED Microscopy

STED microscopy of the protein-coated nanoparticles was performed in a similar way as previously published.^48^ Fluorescent dye, Star Red NHS carbonate (638 nm/655 nm), suitable for STED microscopy was purchased from Abberior and directly used for protein labeling. For the measurement, a solution of BSA and a solution of dye were made at the same molar concentration. After incubation, protein–dye mixture solution was dialyzed with a Slide-A-lyzer Mini Dialysis Device (Thermo Fisher) and redispersed in HEPES buffer (pH 7.4). An Abberior Expertline STED microscope was then used for STED microscopy. The nanoparticles were imaged with a 100× NA 1.4 oil objective. Star Red labeled samples were excited with a 640 nm pulsed laser (40 MHz). The power of the excitation lasers ranged between 5 and 10 mW at the back aperture of the objective. To deplete the fluorescent signals from the dye, a pulsed STED beam of 795 nm at a power ranging from 100 to 500 mW at back aperture applied.

### TEM Measurements

TEM imaging was performed using a JEOL ARM 200F, operated at 200 kV in high angle annular dark field (HAADF) scanning transmission electron microscopy (STEM) mode. TEM sample preparation was performed by placing 4 μL of dispersion on a holey carbon film and allowing it to dry at ambient conditions.

### BEM Simulations

Numerical calculation based on the MNPBEM toolbox^80^ was used to simulate light scattering of a coated single gold nanorod. The simulation was performed using a quasi-static solver. The dielectric function for gold used in the simulation was based on the values measured by the Johnson and Christy model, and the protein layer had various real refractive indices between 1.4 and 1.55. The environment refractive index was set at 1.33 equiv to water. To generate valid results for the coated nanorod with a coated protein layer as thin as 1 nm, fine mesh sizes were used to model the boundaries between a nanorod and protein layer. A plane wave excitation polarized along the longitudinal direction of the nanorod was always used throughout the simulation. The simulated scattering cross sections at different wavelengths were fit with a single Lorentzian function, from which the LSPR wavelengths of the bare and coated nanorods were extracted. The code is freely available at github.com.

## Supporting Information Available

Supplementary figures of time traces and spectroscopy of gold nanorods, numerical modeling, dependence of signal on serum dilution, and figure illustrating the conversion of change in scattered intensity to plasmon shift (PDF)
