# Supplementary material for: Real-Time Optical Tracking of Protein Corona Formation on Single Nanoparticles in Serum
Source: ACS Nano. 2023 Oct 6;17(20):20167–78. doi: 10.1021/acsnano.3c05872 (PMC10604089; doi:10.1021/acsnano.3c05872)
Supplement: Supplementary file 1 — nn3c05872_si_001.pdf [file nn3c05872_si_001.pdf]

Supporting information

Real-time Optical Tracking of Protein Corona Formation on Single Nanoparticles in Serum

Mathias Dolci[1,2], Yuyang Wang[1,2], Sjoerd W. Nooteboom[1,2], Paul E. D. Soto Rodriguez[4], Samuel Sanchez[5,6], Lorenzo Albertazzi[2,3], Peter Zijlstra[1,2]*

1. Department of Applied Physics and Science Education, Eindhoven University of Technology, 5600 MB Eindhoven, The Netherlands

2. Institute for Complex Molecular Systems, Eindhoven University of Technology, 5600 MB Eindhoven, The Netherlands

3. Department of Biomedical Engineering, Eindhoven University of Technology, 5600 MB Eindhoven The Netherlands

4. Departamento de Física, Universidad de la Laguna, C/Astrofísico Francisco Sánchez, s/n.E-38203, Tenerife, Spain

5. Institute for Bioengineering of Catalonia (IBEC), The Barcelona Institute for Science and Technology (BIST), Baldiri Reixac 10-12, 08028 Barcelona, Spain.

6. Institució Catalana de Recerca i Estudis Avançats (ICREA), Passeig de Lluís Companys, 23, 08010 Barcelona, Spain.

*p.zijlstra@tue.nl

Table of contents

Figure S1. Scattering intensity of gold nanorods 3
Figure S2. Spectroscopy of a single gold nanorod 3
Figure S3. Rinsing of the gold nanorods and silica nanoparticles 4
Figure S4. Simulation of the gold nanorod with different thicknesses of PC 4
Figure S5. Simulation of the silica nanoparticle with different thicknesses of PC 5
Figure S6. Plasmon shift of gold nanorods for different FBS concentrations 5
Figure S7. Scattering cross section of gold nanorod before and after PC formation 6
Figure S8. Linewidth variation after PC formation 6

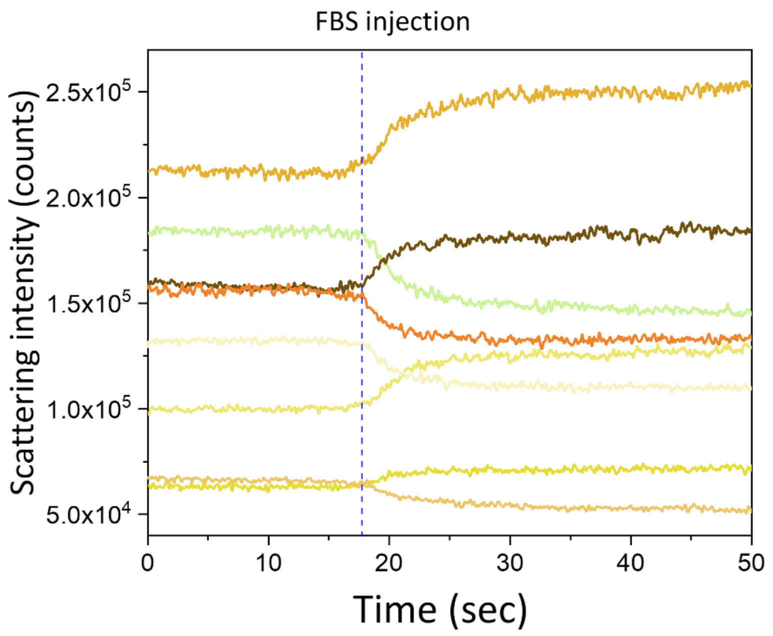

Figure S1. Scattering intensity of gold nanorods. Timetrace of few single gold nanorods in the field of view. The vertical line correspond to the injection of undiluted FBS.

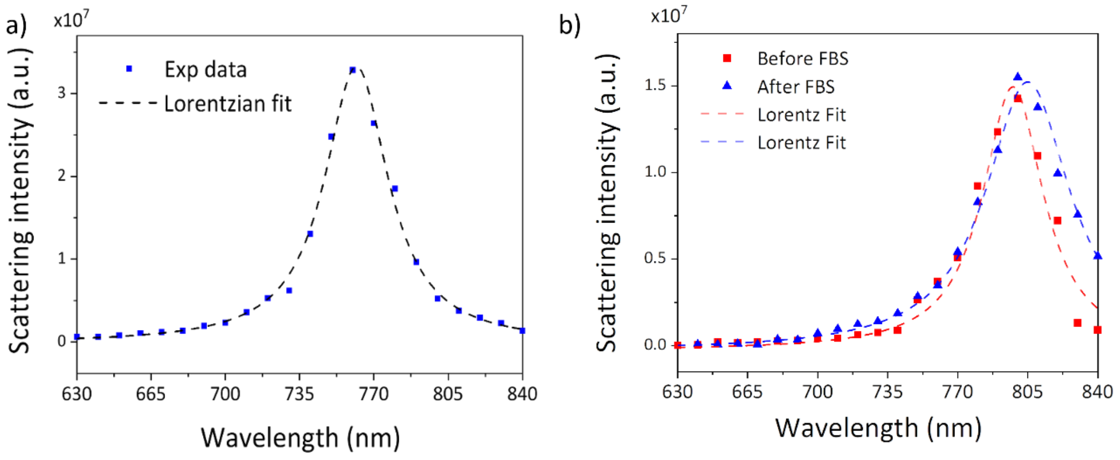

Figure S2. Spectroscopy of a single gold nanorod. a) Spectroscopy of a single AuNR in the field of view. Each intensity have been determined for wavelength ranging from 600 nm to 840 nm, the spectrum has been fitted with a Lorentzian function to determine the central wavelength and the linewidth. b) Spectroscopy of a single AuNR before and after injection of FBS.

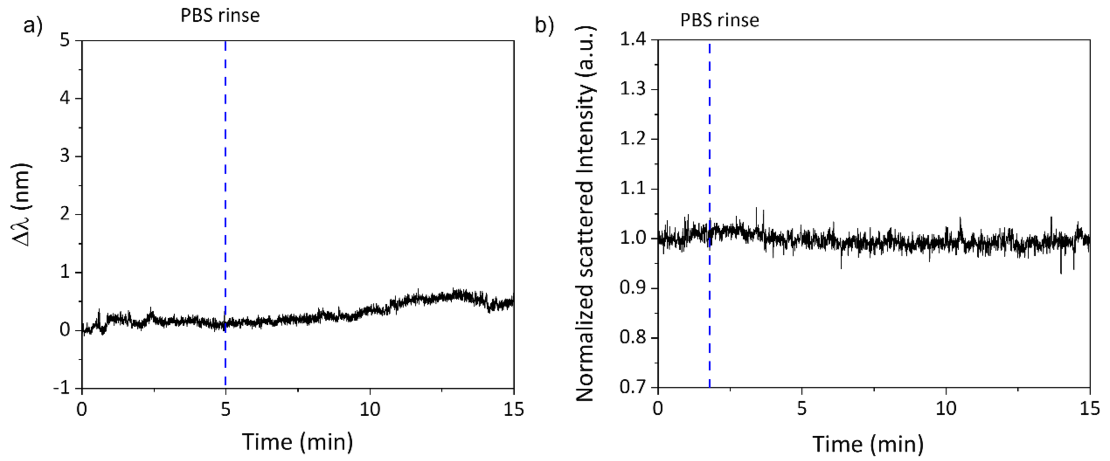

Figure S3. Rinsing of the gold nanorods and silica nanoparticles. Averaged timetrace of all the single SiO_2 nanoparticles in the field of view. The vertical line correspond to the rinse with PBS/EG matching refractive index.

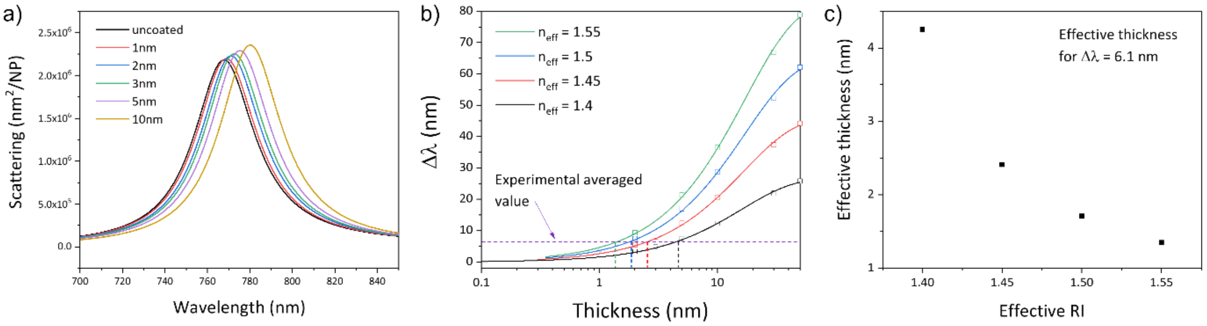

Figure S4. Simulation of the gold nanorod with different thicknesses of PC. a) Simulation of the scattering cross section of a 20x75nm gold nanorod with different thickness of organic layer corresponding to PC layer with a refractive index n=1.4. b) Spectral shift plotted versus the thickness of PC and for different refractive indices and c) effective thickness corresponding to the spectral shift obtained in the experimental data (horizontal dashed line in b) for different refractive indices.

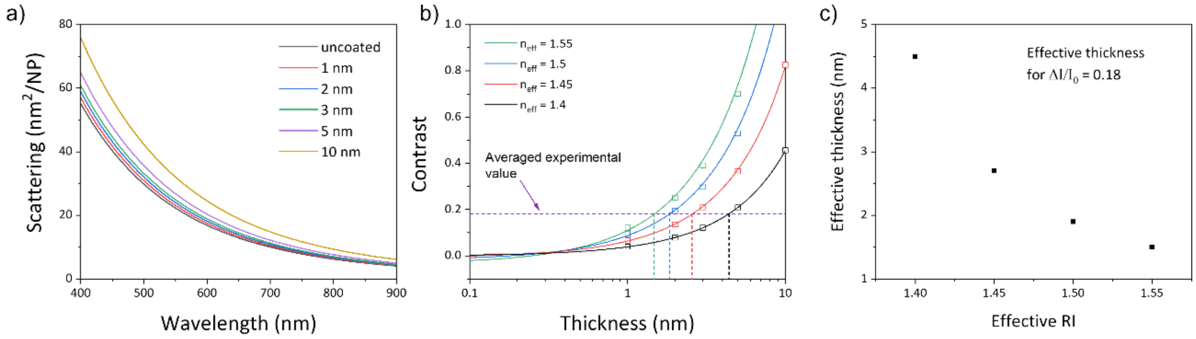

Figure S5. Simulation of the silica nanoparticle with different thicknesses of PC. a) Simulation of the scattering cross section of a 150nm silica nanoparticle with different thickness of organic layer corresponding to PC layer with a refractive index n=1.4. b) Contrast plotted versus the thickness of PC and for different refractive indices and c) effective thickness corresponding to the contrast obtained in the experimental data (horizontal dashed line in b) for different refractive indices.

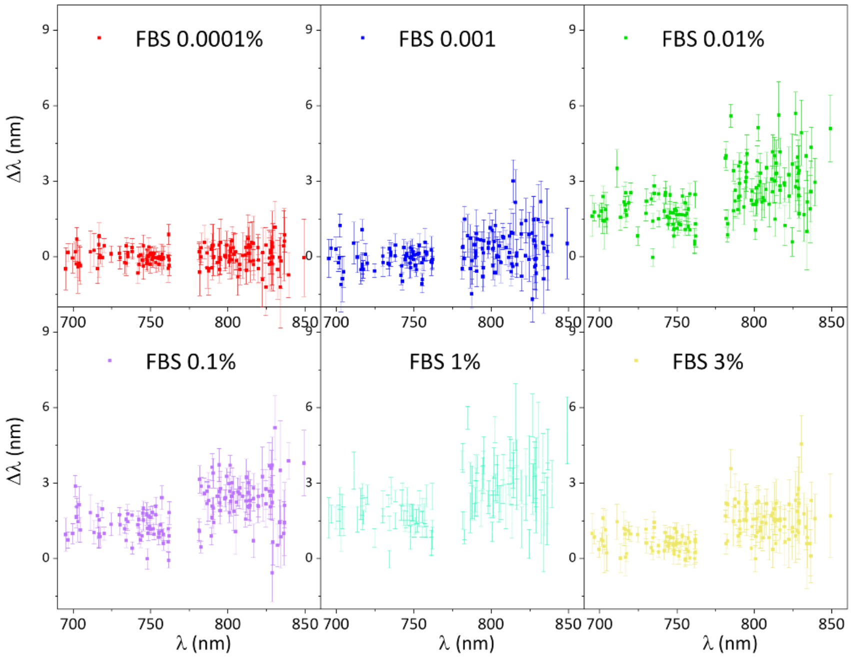

Figure S6. Plasmon shift of gold nanorods for different FBS concentrations. Plasmon shifts plotted as function of the resonance wavelength for different concentration of FBS sequentially injected.

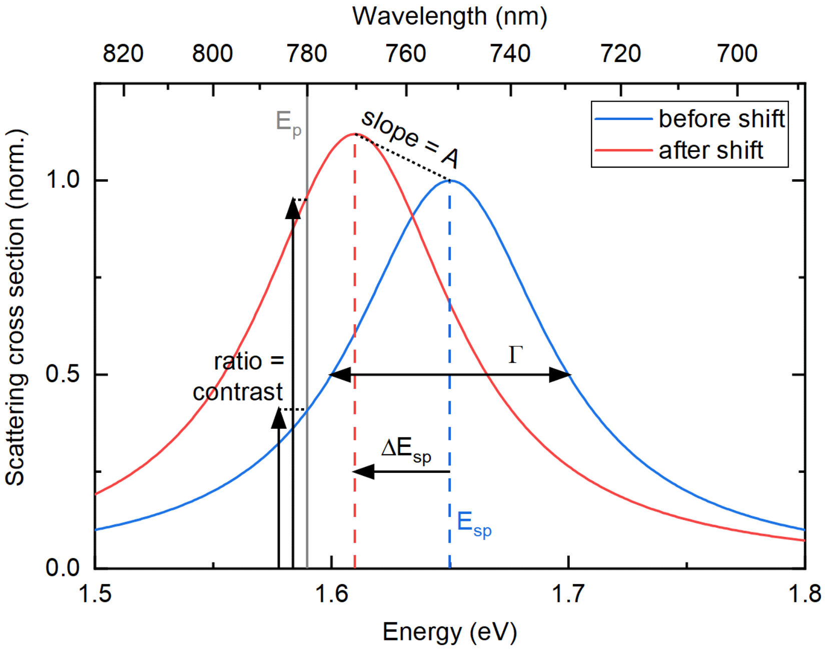

Figure S7. Scattering cross section of gold nanorod before and after PC formation. Illustration of the quantities used in the derivation for the conversion of the change in scattered intensity to plasmon shift for the AuNRs

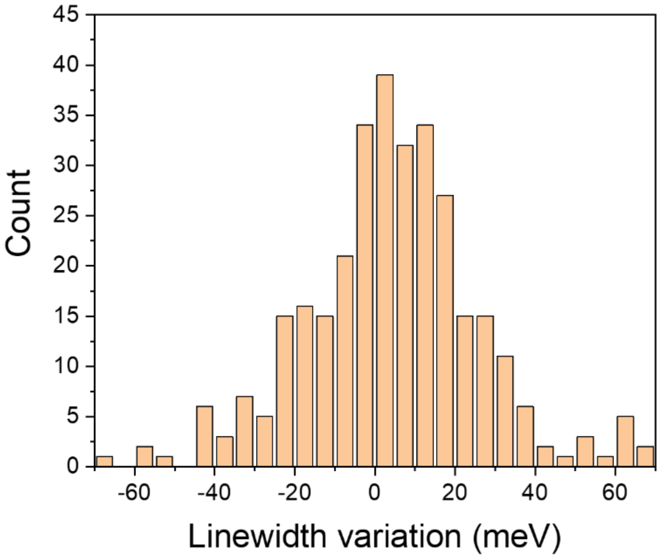

Figure S8. Linewidth variation after PC formation. Histogram of the change in the linewidth of the spectra of single particles after adsorption of the proteins
